# Supplementary material for: Predictive biomarkers for the responsiveness of recurrent glioblastomas to activated killer cell immunotherapy
Source: Cell Biosci. 2023 Jan 24;13:17. doi: 10.1186/s13578-023-00961-4 (PMC9875464; doi:10.1186/s13578-023-00961-4)
Supplement: Supplementary file 1 — Additional file 1: Figure S1. The expression patterns of ligands of various NK-activating receptors. (A) Heatmap for 40 genes related to immune response including NK-activating receptors from 12 patients. Expression levels were aligned according to functional annotation groups. Patients were grouped by treatment response. Responders are in green and non-responders are in red. Genes with a p-value< 0.05 are denoted with an asterisk (*). The mRNA expression levels are represented as colors from red (up-regulated, z-score 4) to blue (down-regulated, z-score −4). (B) Correlation plots of immune-cell receptors and their ligands in responders and non-responders. Pearson’s correlation coefficient scores are represented as colors from red (1.0) to blue (−1.0). Figure S2. The correlation of CD8+ and CD68+ cells in the tumor area. (A) The dot plots between CD8+ cells and CD68+ cells in the tumor area, the trend line, and confidence interval (gray area) between them. In responders, they are significantly correlated (Pearson’s correlation coefficient = 0.95, p = 0.013), but in non-responders, they are not (Pearson’s correlation coefficient = 0.47, p = 0.29). (B) Density map of high-density areas of CD8+ and CD68+ cells. In responders, the high-density areas of CD8+ and CD68+cells seemed to be located more frequently in the tumor tissue rather than the non-tumor tissue, with similar distribution patterns within the same piece of tissue. This suggests that the cases with close interaction of CD8+ and CD68+ cells in the tumor might respond better to NK cell therapy. In contrast, non-responders showed somewhat nonspecific distribution patterns of high-density CD8+ and CD68+ cell areas. Only a few areas showed similar distributions in the same piece of tissue from non-responders. (C) The correlation plots between protein expression by immunohistochemistry and the mRNA expression of each gene by NanoString in responders and in non-responders. In responders, they are significantly cor [file 13578_2023_961_MOESM1_ESM.docx]

**Additional figures
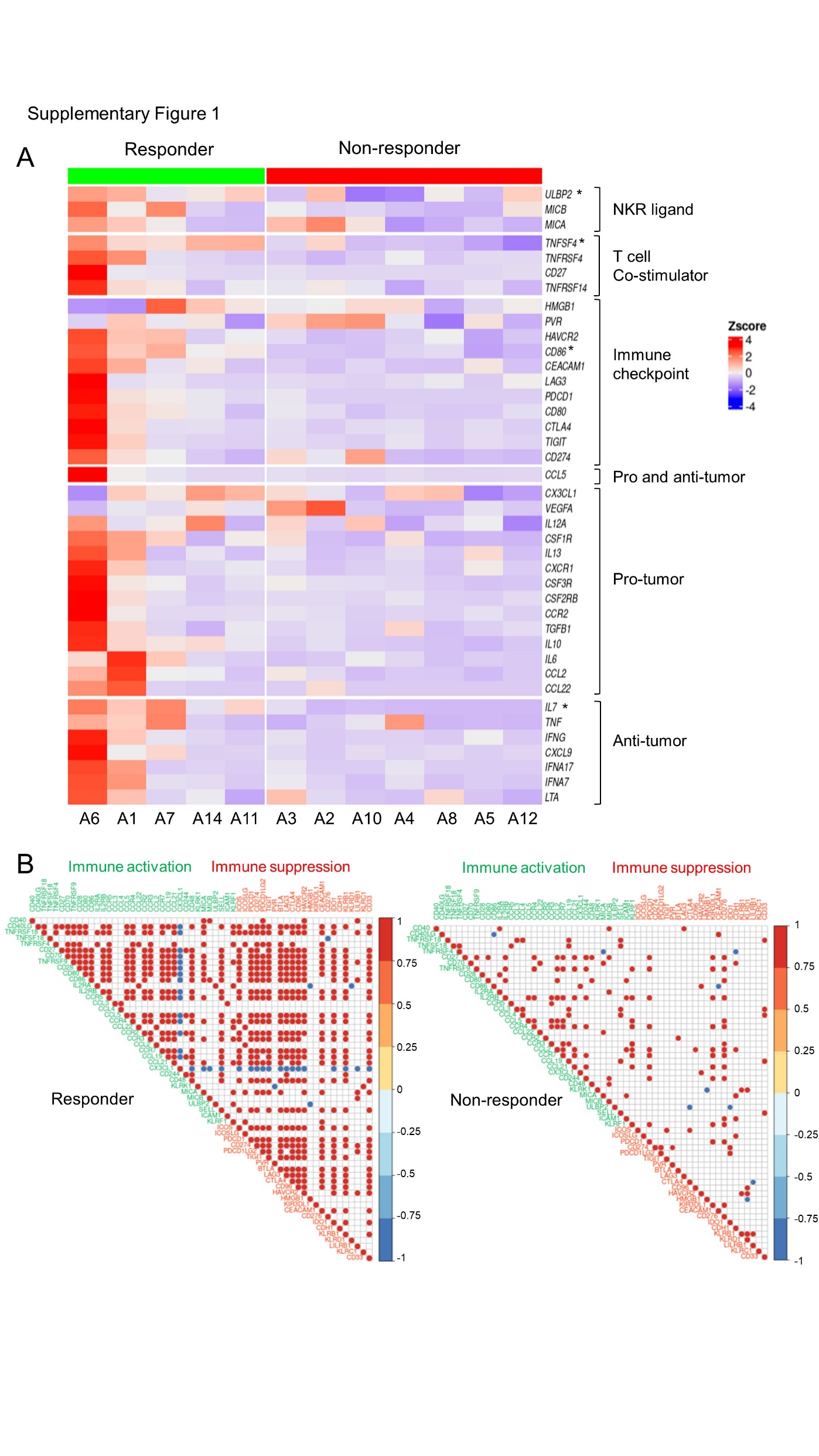
**

**Figure S1. The expression patterns of ligands of various NK-activating receptors.** (A) Heatmap for 40 genes related to immune response including NK-activating receptors from 12 patients. Expression levels were aligned according to functional annotation groups. Patients were grouped by treatment response. Responders are in green and non-responders are in red. Genes with a *p*-value < 0.05 are denoted with an asterisk (*). The mRNA expression levels are represented as colors from red (up-regulated, z-score 4) to blue (down-regulated, z-score −4). (B) Correlation plots of immune-cell receptors and their ligands in responders and non-responders. Pearson’s correlation coefficient scores are represented as colors from red (1.0) to blue (−1.0).

**
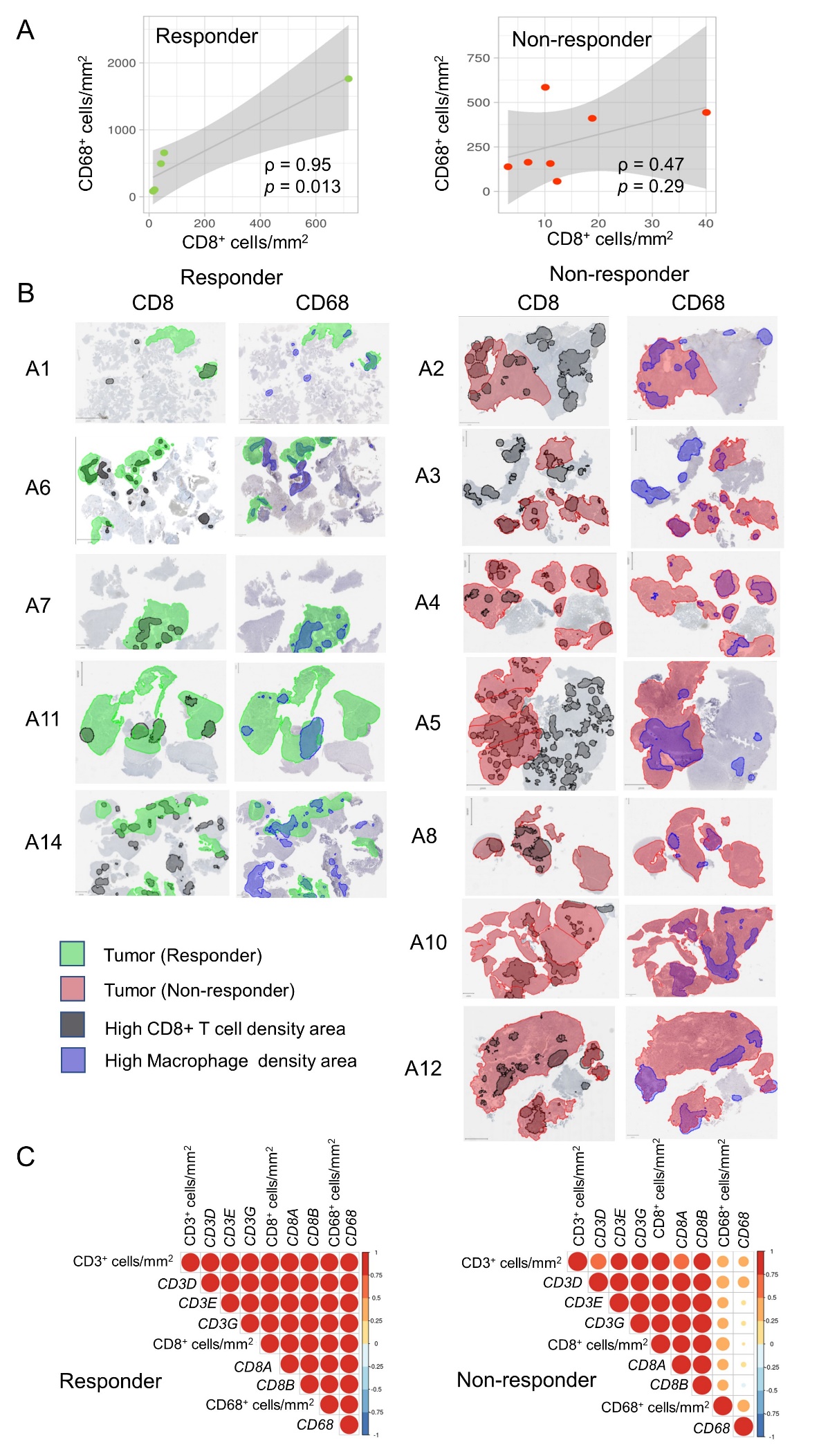
 Figure S2. The correlation of CD8^+^ and CD68^+^ cells in the tumor area.** (A) The dot plots between CD8**^+^** cells and CD68**^+^** cells in the tumor area, the trend line, and confidence interval (gray area) between them. In responders, they are significantly correlated (Pearson’s correlation coefficient = 0.95, *p* = 0.013), but in non-responders, they are not (Pearson’s correlation coefficient = 0.47, *p* = 0.29). (B) Density map of high-density areas of CD8^+^ and CD68^+^ cells. In responders, the high-density areas of CD8^+^ and CD68^+^ cells seemed to be located more frequently in the tumor tissue rather than the non-tumor tissue, with similar distribution patterns within the same piece of tissue. This suggests that the cases with close interaction of CD8^+^ and CD68^+^ cells in the tumor might respond better to NK cell therapy. In contrast, non-responders showed somewhat nonspecific distribution patterns of high-density CD8^+^ and CD68^+^ cell areas. Only a few areas showed similar distributions in the same piece of tissue from non-responders. (C) The correlation plots between protein expression by immunohistochemistry and the mRNA expression of each gene by NanoString in responders and in non-responders. In responders, they are significantly correlated, but in non-responders, CD68 were not significantly correlated to others.


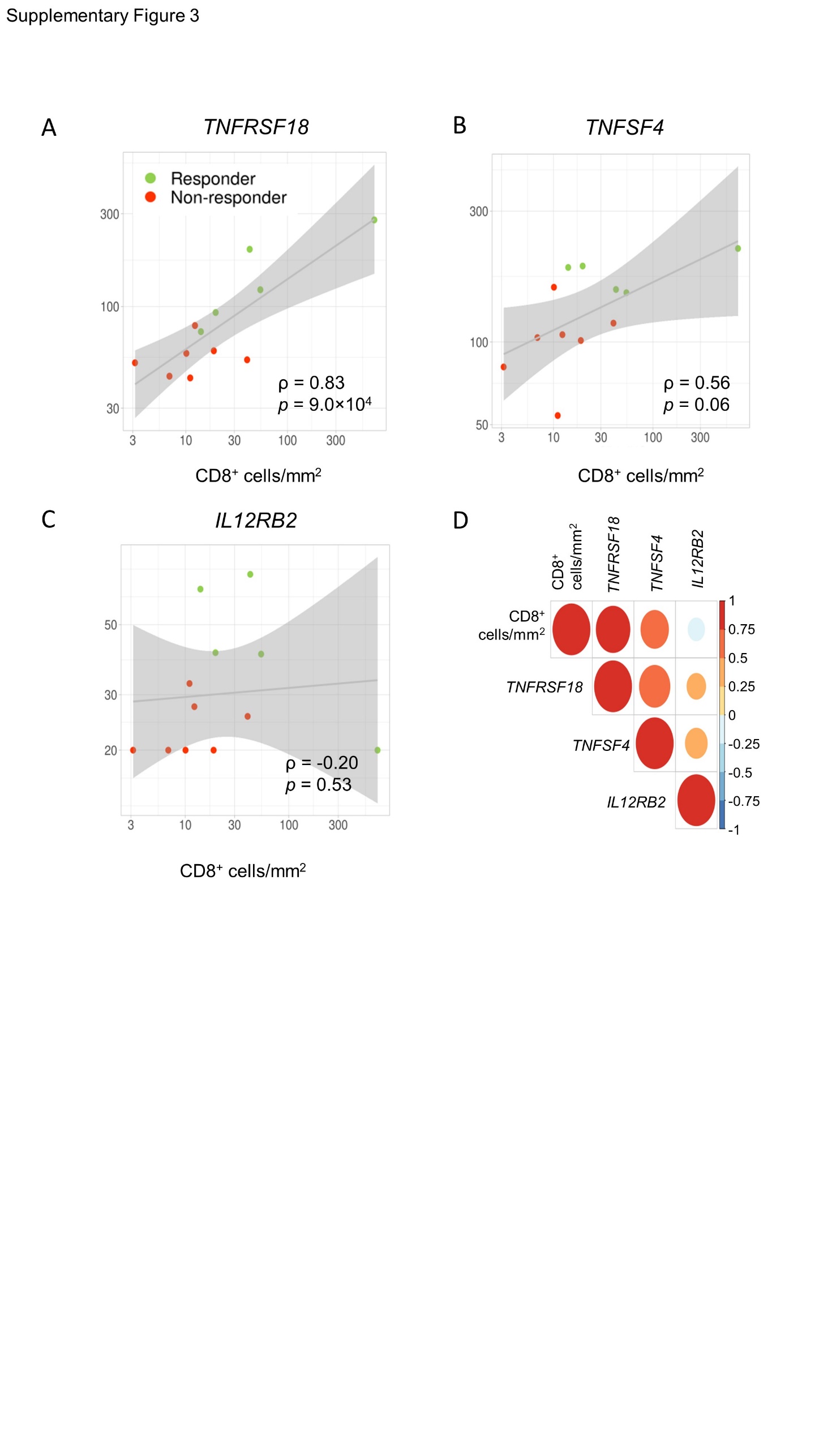


**Figure S3. The correlation of CD8^+^ cytotoxic T cells by immunohistochemical staining and mRNA expression of three genes by Nanostring.** The dot plots show the correlation between CD8**^+^** cells in the tumor area and mRNA expression levels of *TNFRSF18*, *TNFSF4*, and *IL12RB2* according to the NanoString analysis. (A) *TNFRSF18* and was significantly correlated with CD8**^+^** cells in the tumor area (Pearson’s correlation coefficient = 0.83, *p* = 9.0 × 10^4^). (B) *TNFSF4* was also correlated with CD8**^+^** cells in the tumor area (Pearson’s correlation coefficient = 0.56, *p* = 0.06). (C) *IL12RB2* was not correlated with CD8**^+^** cells in the tumor area (Pearson’s correlation coefficient = −0.20, *p* = 0.53). (D) The correlation plot also shows the same pattern between these genes and CD8^+^ cells. Pearson’s correlation coefficient scores are represented as colors from red (1.0) to blue (−1.0). The size of the circle represents the statistical significance; the bigger the circle, the greater the significance.
