# Supplementary material for: Predictive biomarkers for the responsiveness of recurrent glioblastomas to activated killer cell immunotherapy
Source: Cell Biosci. 2023 Jan 24;13:17. doi: 10.1186/s13578-023-00961-4 (PMC9875464; doi:10.1186/s13578-023-00961-4)
Supplement: Supplementary file 3 — Additional file 3: Table S1. Immunohistochemical stain reagents and staining conditions. Table S2. Prior treatment information of each patient. Table S3. Seven signature scores of functional annotation groups discriminating responder and non-responder patients. Table S4. 35 differentially expressed genes between responders and non-responders. Table S5. 64 significantly associated genes to therapy's response. Table S6. The prediction performance of random forest models. Table S7. The mean number of immune cells per mm2 in tumor by immunohistochemistry. [file 13578_2023_961_MOESM3_ESM.docx]

**Additional tables**

**Table S1. Immunohistochemical stain reagents and staining conditions.**

| Antibody | Company | Antigen retrieval time (min) | Antibody application time (min) |
| --- | --- | --- | --- |
| CD3(2GV6) | Roche Diagnostics | 36 | 32 |
| CD8(SP57) | Roche Diagnostics | 64 | 32 |
| CD68(KP-1) | Roche Diagnostics | 64 | 32 |

**Table S2. Prior treatment information of each patient.**

| Patients | Response | Prior treatment | Re-RTx | IDH1 status | Salvage chemo-Tx |
| --- | --- | --- | --- | --- | --- |
| A1 | R | CCRTx + 6cycle TMZ | Yes | Wildtype | BCNU |
| A2 | NR | CCRTx + 6cycle TMZ | Yes | Wildtype | BCNU |
| A3 | NR | CCRTx + 6cycle TMZ | Yes | Wildtype | BCNU |
| A4 | NR | CCRTx + 6cycle TMZ | Yes | Wildtype | BCNU |
| A5 | NR | CCRTx + 6cycle TMZ | Yes | Wildtype | BCNU |
| A6 | R | CCRTx + 6cycle TMZ | Yes | Wildtype | BCNU |
| A7 | R | CCRTx + 6cycle TMZ | Yes | Wildtype | BCNU |
| A8 | NR | CCRTx + 6cycle TMZ | Yes | Mutation | BCNU |
| A9 | NR | CCRTx + 6cycle TMZ | Yes | Wildtype | Bevacizumab |
| A10 | NR | CCRTx + 6cycle TMZ | Yes | Wildtype | ACNU |
| A11 | R | CCRTx + 6cycle TMZ | Yes | Wildtype | ACNU |
| A12 | NR | CCRTx + 6cycle TMZ | Yes | Wildtype | ACNU |
| A13 | NR | CCRTx + 6cycle TMZ | No | Wildtype | ACNU |
| A14 | R | CCRTx + 6cycle TMZ | Yes | Wildtype | ACNU |

**CCRTx + 6cycle TMZ; Concurrent Chemo-Radiation Treatment (with Temozolomide) + 6cycle Temozolomide, Re-RTx**; Re-irradiation, **Tx**; Treatment; R; Responder, NR; Non-responder.

**Table S3. Seven signature scores of functional annotation groups discriminating responder and non-responder patients.**

| Function | AUC | t-test p-value | adjusted p-value |
| --- | --- | --- | --- |
| Senescence | 0.886 | 0.026 | 0.268 |
| Cell cycle | 0.914 | 0.009 | 0.185 |
| Chemokines | 0.829 | 0.074 | 0.357 |
| Regulation | 0.829 | 0.106 | 0.357 |
| Cytokines | 0.829 | 0.119 | 0.357 |
| TNF superfamily | 0.800 | 0.094 | 0.357 |
| Interleukins | 0.829 | 0.082 | 0.357 |

AUC; Area Under a ROC Curve.

**Table S4. 35 differentially expressed genes between responders and non-responders.**

| Gene symbol | AUC | t-test  p-value | adjusted  p-value | average expression of responder | average expression of non-responders | Fold change | DEG |
| --- | --- | --- | --- | --- | --- | --- | --- |
| *TNFSF4* | 0.94 | 0.0014 | 0.49 | 180.41 | 103.17 | 1.75 | up |
| *IL34* | 1.00 | 0.0036 | 0.49 | 180.06 | 63.20 | 2.85 | up |
| *NOTCH1* | 0.97 | 0.0082 | 0.49 | 1629.68 | 2508.00 | 0.65 | down |
| *CD47* | 0.91 | 0.0091 | 0.49 | 5496.61 | 3558.92 | 1.54 | up |
| *NFKB1* | 0.94 | 0.0098 | 0.49 | 340.64 | 226.26 | 1.51 | up |
| *HLA-G* | 0.97 | 0.0102 | 0.49 | 2901.93 | 1304.42 | 2.22 | up |
| *FOS* | 0.94 | 0.0116 | 0.49 | 29964.84 | 13190.11 | 2.27 | up |
| *IRF2* | 0.89 | 0.0130 | 0.49 | 1018.54 | 767.44 | 1.33 | up |
| *IL7* | 1.00 | 0.0157 | 0.49 | 65.65 | 22.37 | 2.94 | up |
| *PECAM1* | 1.00 | 0.0164 | 0.49 | 2548.55 | 1168.98 | 2.18 | up |
| *APP* | 0.91 | 0.0194 | 0.49 | 27727.14 | 17885.43 | 1.55 | up |
| *TICAM2* | 0.97 | 0.0203 | 0.49 | 308.30 | 234.56 | 1.31 | up |
| *ITGA1* | 0.94 | 0.0209 | 0.49 | 1221.72 | 693.15 | 1.76 | up |
| *F13A1* | 1.00 | 0.0221 | 0.49 | 2011.60 | 395.02 | 5.09 | up |
| *TNFSF18* | 0.94 | 0.0259 | 0.49 | 143.85 | 49.31 | 2.92 | up |
| *CD86* | 1.00 | 0.0261 | 0.49 | 390.29 | 156.01 | 2.50 | up |
| *CD58* | 0.83 | 0.0292 | 0.49 | 1011.33 | 777.55 | 1.30 | up |
| *UBC* | 0.86 | 0.0308 | 0.49 | 84150.48 | 66348.47 | 1.27 | up |
| *C1S* | 0.89 | 0.0313 | 0.49 | 2519.39 | 1421.60 | 1.77 | up |
| *C3* | 0.97 | 0.0315 | 0.49 | 13865.90 | 5139.26 | 2.70 | up |
| *STAT4* | 0.94 | 0.0317 | 0.49 | 139.98 | 45.86 | 3.05 | up |
| *CCL3L1* | 0.91 | 0.0323 | 0.49 | 10675.48 | 3070.79 | 3.48 | up |
| *MAVS* | 0.91 | 0.0323 | 0.49 | 1343.46 | 2129.56 | 0.63 | down |
| *MEF2C* | 0.89 | 0.0354 | 0.49 | 3012.08 | 1839.02 | 1.64 | up |
| *EGR2* | 0.86 | 0.0380 | 0.49 | 955.87 | 490.28 | 1.95 | up |
| *CASP1* | 0.91 | 0.0388 | 0.49 | 1024.77 | 466.99 | 2.19 | up |
| *SMPD3* | 0.89 | 0.0408 | 0.49 | 69.50 | 50.14 | 1.39 | up |
| *CYLD* | 0.89 | 0.0422 | 0.49 | 2462.42 | 1607.89 | 1.53 | up |
| *ICAM2* | 0.97 | 0.0458 | 0.49 | 405.57 | 201.50 | 2.01 | up |
| *REL* | 0.89 | 0.0459 | 0.49 | 681.46 | 363.99 | 1.87 | up |
| *MAP3K5* | 0.86 | 0.0459 | 0.49 | 2720.01 | 1558.23 | 1.75 | up |
| *ICAM1* | 0.86 | 0.0461 | 0.49 | 2065.60 | 831.59 | 2.48 | up |
| *MAPK3* | 0.80 | 0.0466 | 0.49 | 2334.33 | 1843.12 | 1.27 | up |
| *CXCL5* | 0.83 | 0.0483 | 0.49 | 95.45 | 46.30 | 2.06 | up |
| *ULBP2* | 0.83 | 0.0494 | 0.49 | 113.94 | 75.15 | 1.52 | up |

AUC; Area Under a ROC Curve, Fold change; responder/non-responder, DEG; up or down in responder compared to non-responder.

**Table S5. 64 significantly associated genes to therapy's response.**

| Gene symbol | AUC | fold change | OS  p-value | OS adjusted p-value | PFS  p-value | PFS adjusted p-value | Functional  category |
| --- | --- | --- | --- | --- | --- | --- | --- |
| *APP* | 0.91 | 1.55 | 0.004 | 0.130 | 0.006 | 0.104 |  |
| *C3* | 0.97 | 2.70 | 0.010 | 0.168 | 0.006 | 0.104 | Regulation |
| *CASP1* | 0.91 | 2.19 | 0.004 | 0.130 | 0.002 | 0.088 |  |
| *CCL19* | 0.89 | 9.82 | 0.047 | 0.239 | 0.081 | 0.360 | Chemokines, regulation |
| *CCL3* | 0.89 | 2.84 | 0.036 | 0.229 | 0.105 | 0.360 | Chemokines, regulation |
| *CCL3L1* | 0.91 | 3.48 | 0.036 | 0.229 | 0.105 | 0.360 | Cytokines |
| *CCL4* | 0.91 | 3.62 | 0.036 | 0.229 | 0.105 | 0.360 | Chemokines, regulation |
| *CD3E* | 0.94 | 9.17 | 0.014 | 0.168 | 0.003 | 0.088 | B cell functions, cell functions, T-cell functions |
| *CD5* | 0.97 | 3.93 | 0.014 | 0.168 | 0.003 | 0.088 | B cell functions, regulation, T cell functions |
| *CD83* | 0.91 | 2.70 | 0.016 | 0.168 | 0.056 | 0.360 |  |
| *CD86* | 1.00 | 2.50 | 0.021 | 0.182 | 0.011 | 0.178 | B cell functions, macrophage functions, regulation, T cell functions |
| *CD97* | 0.86 | 1.47 | 0.004 | 0.130 | 0.002 | 0.088 |  |
| *CEACAM1* | 0.86 | 2.26 | 0.041 | 0.234 | 0.146 | 0.413 | Adhesion |
| *CMKLR1* | 0.89 | 3.04 | 0.035 | 0.229 | 0.088 | 0.360 | Chemokines |
| *COL3A1* | 0.89 | 6.36 | 0.022 | 0.182 | 0.038 | 0.322 | Regulation |
| *CXCL1* | 0.91 | 3.25 | 0.041 | 0.234 | 0.146 | 0.413 | Chemokines, regulation |
| *CXCL12* | 0.89 | 2.48 | 0.001 | 0.130 | 0.000 | 0.088 | Chemokines |
| *CYFIP2* | 0.86 | 1.76 | 0.096 | 0.306 | 0.011 | 0.178 |  |
| *EGR2* | 0.86 | 1.95 | 0.001 | 0.130 | 0.000 | 0.088 | Regulation |
| *F13A1* | 1.00 | 5.09 | 0.014 | 0.168 | 0.003 | 0.088 | Cell functions |
| *FCGR2A* | 0.94 | 2.30 | 0.014 | 0.168 | 0.003 | 0.088 | Transporter functions |
| *FOS* | 0.94 | 2.27 | 0.016 | 0.168 | 0.105 | 0.360 |  |
| *HLA-DRA* | 1.00 | 2.75 | 0.014 | 0.168 | 0.003 | 0.088 | Antigen processing |
| *HLA-G* | 0.97 | 2.22 | 0.004 | 0.130 | 0.002 | 0.088 | Regulation |
| *ICAM2* | 0.97 | 2.01 | 0.021 | 0.182 | 0.011 | 0.178 | Adhesion, regulation |
| *IL10* | 1.00 | 3.64 | 0.010 | 0.168 | 0.006 | 0.104 | Interleukins |
| *IL10RA* | 0.97 | 2.57 | 0.010 | 0.168 | 0.006 | 0.104 | Cytokines |
| *IL12RB2* | 0.86 | 2.01 | 0.114 | 0.350 | 0.016 | 0.241 | Cytokines, NK cell functions, T cell functions |
| *IL18* | 0.91 | 2.41 | 0.005 | 0.130 | 0.023 | 0.270 | Interleukins, NK cell functions, T cell functions |
| *IL1B* | 0.86 | 3.23 | 0.016 | 0.168 | 0.056 | 0.360 | Chemokines, cytokines, interleukins, pathogen defense, regulation |
| *IL34* | 1.00 | 2.85 | 0.014 | 0.168 | 0.003 | 0.088 | Interleukins |
| *IL6* | 0.86 | 5.00 | 0.016 | 0.168 | 0.056 | 0.360 | Interleukins |
| *IL7* | 1.00 | 2.94 | 0.014 | 0.168 | 0.003 | 0.088 | Interleukins |
| *IL7R* | 0.94 | 4.13 | 0.014 | 0.168 | 0.003 | 0.088 | Cytokines |
| *IL8* | 0.89 | 3.14 | 0.036 | 0.229 | 0.105 | 0.360 | Chemokines, cytokines, interleukins, Pathogen defense, regulation |
| *IRF2* | 0.89 | 1.33 | 0.004 | 0.130 | 0.002 | 0.088 | Chemokines, regulation |
| *ITGA1* | 0.94 | 1.76 | 0.014 | 0.168 | 0.003 | 0.088 | Adhesion, NK cell functions, T cell functions |
| *ITK* | 0.86 | 4.96 | 0.047 | 0.239 | 0.081 | 0.360 |  |
| *LAMP3* | 0.97 | 3.65 | 0.014 | 0.168 | 0.003 | 0.088 | Cell functions |
| *LCK* | 0.89 | 5.92 | 0.047 | 0.239 | 0.081 | 0.360 | Regulation, T cell functions |
| *LILRA1* | 0.89 | 2.21 | 0.035 | 0.229 | 0.088 | 0.360 | Regulation |
| *MAP3K5* | 0.86 | 1.75 | 0.041 | 0.234 | 0.134 | 0.406 |  |
| *MAP4K2* | 0.86 | 1.28 | 0.041 | 0.234 | 0.146 | 0.413 |  |
| *MEF2C* | 0.89 | 1.64 | 0.041 | 0.234 | 0.016 | 0.241 |  |
| *MPPED1* | 0.86 | 2.27 | 0.004 | 0.130 | 0.006 | 0.104 | Cell functions |
| *NEFL* | 0.86 | 3.52 | 0.072 | 0.306 | 0.026 | 0.290 | Cell functions |
| *NLRP3* | 0.86 | 2.28 | 0.005 | 0.130 | 0.023 | 0.270 |  |
| *NOTCH1* | 0.97 | 0.65 | 0.004 | 0.130 | 0.002 | 0.088 | Regulation |
| *PDCD1* | 0.97 | 2.51 | 0.014 | 0.168 | 0.003 | 0.088 | Regulation |
| *PECAM1* | 1.00 | 2.18 | 0.014 | 0.168 | 0.003 | 0.088 | Transporter functions |
| *PSMB8* | 0.91 | 1.58 | 0.004 | 0.130 | 0.002 | 0.088 | Chemokines |
| *PTGS2* | 0.91 | 4.64 | 0.036 | 0.229 | 0.105 | 0.360 | Cytokines |
| *PTPRC* | 0.97 | 2.72 | 0.014 | 0.168 | 0.003 | 0.088 | B cell functions, T cell functions |
| *SIGIRR* | 0.89 | 2.44 | 0.016 | 0.168 | 0.053 | 0.360 |  |
| *SMPD3* | 0.89 | 1.39 | 0.014 | 0.168 | 0.003 | 0.088 | Cell functions |
| *STAT4* | 0.94 | 3.05 | 0.004 | 0.130 | 0.006 | 0.104 | Chemokines, regulation, T cell functions |
| *TAP1* | 0.86 | 1.58 | 0.004 | 0.130 | 0.002 | 0.088 | Antigen processing |
| *THBD* | 0.89 | 2.17 | 0.010 | 0.168 | 0.006 | 0.104 | Leukocyte functions |
| *TICAM2* | 0.97 | 1.31 | 0.010 | 0.168 | 0.006 | 0.104 |  |
| *TLR1* | 0.86 | 1.84 | 0.035 | 0.229 | 0.088 | 0.360 | Microglial functions, TLR |
| *TNFRSF18* | 0.97 | 2.76 | 0.001 | 0.130 | 0.000 | 0.088 | TNF superfamily |
| *TNFRSF1B* | 0.89 | 2.86 | 0.005 | 0.130 | 0.023 | 0.270 | Chemokines, TNF superfamily |
| *TNFSF18* | 0.94 | 2.92 | 0.016 | 0.168 | 0.105 | 0.360 | B cell functions, cell functions, T cell functions, TNF superfamily |
| *TNFSF4* | 0.94 | 1.75 | 0.010 | 0.168 | 0.006 | 0.104 | Chemokines, TNF superfamily |

AUC; Area Under a ROC Curve), Fold change (responder/non-responder), OS; overall survival, PFS: progression-free survival.

**Table S6. The prediction performance of random forest models.**

| Random forest model | OOB error rate | Sensitivity | Specificity | # of genes | Gene list |
| --- | --- | --- | --- | --- | --- |
| TNF superfamily | 0.0% | 1.00 | 1.00 | 3 | *IL12RB2, TNFRSF18, TNFSF4* |
| Regulators of T cell activation | 8.3% | 0.80 | 1.00 | 2 | *CD86, LCK* |
| Antigen processing | 8.3% | 1.00 | 0.86 | 2 | *HLA-DRA, TAP1* |
| Adaptive immune response | 8.3% | 1.00 | 0.86 | 11 | *CD3E, CD5, CD86, HLA-DRA, IL12RB2, IL7, IL7R, ITK, PDCD1, STAT4, TAP1* |
| Innate immune response | 8.3% | 0.80 | 1.00 | 14 | *APP, C3, CASP1, CYFIP2, FOS, IL1B, IL34, IL8, MAP3K5, MAP4K2, NLRP3, SIGIRR, TICAM2, TLR1* |
| Inflammation | 16.7% | 0.80 | 0.86 | 4 | *IL1B, IL18, NOTCH1, TLR1* |
| Cytokines | 16.7% | 0.80 | 0.86 | 6 | *CCL3L1, IL10RA, IL12RB2, IL1B, IL7R, IL8* |
| Interleukins | 16.7% | 0.60 | 1.00 | 7 | *IL10, IL18, IL1B, IL34, IL6, IL7, IL8* |
| Adhesion | 25.0% | 0.60 | 0.86 | 3 | *CEACAM1, ICAM2, ITGA1* |
| NK cell functions | 25.0% | 0.60 | 0.86 | 3 | *IL12RB2, IL18, ITGA1* |
| B cell functions | 25.0% | 0.60 | 0.86 | 5 | *CD3E, CD5, CD86, PTPRC, TNFSF18* |
| Humoral immune response | 25.0% | 0.60 | 0.86 | 6 | *CCL3, CD83, IL6, IL7, MEF2C, PDCD1* |
| Inflammasome pathway | 25.0% | 0.60 | 0.86 | 7 | *CASP1, NLRP3, IL18, IL1B, NOTCH1, TLR1, TICAM2* |
| T cell functions | 25.0% | 0.60 | 0.86 | 10 | *CD3E, CD5, CD86, IL12RB2, IL18, ITGA1, LCK, PTPRC, STAT4, TNFSF18* |
| Chemokines | 25.0% | 0.80 | 0.71 | 13 | *CCL19, CCL3, CCL4, CMKLR1, CXCL1, CXCL12, IL1B, IL8, IRF2, PSMB8, STAT4, TNFRSF1B, TNFSF4* |
| All 64 genes | 25.0% | 0.60 | 0.86 | 64 | *APP, C3, CASP1, CCL19, CCL3, CCL3L1, CCL4, CD3E, CD5, CD83, CD86, CD97, CEACAM1, CMKLR1, COL3A1, CXCL1, CXCL12, CYFIP2, EGR2, F13A1, FCGR2A, FOS, HLA-DRA, HLA-G, ICAM2, IL10, IL10RA, IL12RB2, IL18, IL1B, IL34, IL6, IL7, IL7R, IL8, IRF2, ITGA1, ITK, LAMP3, LCK, LILRA1, MAP3K5, MAP4K2, MEF2C, MPPED1, NEFL, NLRP3, NOTCH1, PDCD1, PECAM1, PSMB8, PTGS2, PTPRC, SIGIRR, SMPD3, STAT4, TAP1, THBD, TICAM2, TLR1, TNFRSF18, TNFRSF1B, TNFSF18, TNFSF4* |

OOB error rate; Out-of-bag error rate.

**Table S7. The mean number of immune cells per mm^2^ in tumor by immunohistochemistry.**

| Patients | Response | CD3  (cells/mm^2^) | CD8  (cells/mm^2^) | CD68  (cells/mm^2^) |
| --- | --- | --- | --- | --- |
| A1 | R | 12.4 | 42.47 | 495.68 |
| A2 | NR | 26.5 | 10.09 | 585.23 |
| A3 | NR | 43.9 | 40.07 | 443.79 |
| A4 | NR | Not available | 12.28 | 56.77 |
| A5 | NR | 15.8 | 3.15 | 138.8 |
| A6 | R | 786.3 | 717.65 | 1762.98 |
| A7 | R | 72.3 | 54.07 | 657.44 |
| A8 | NR | 28.5 | 6.88 | 164.01 |
| A10 | NR | 28.6 | 18.82 | 410.9 |
| A11 | R | 13.9 | 19.63 | 106.15 |
| A12 | NR | 32.3 | 11.02 | 156.8 |
| A14 | R | 29.0 | 14.04 | 83.6 |

R; Responder, NR; Non-responder.
